# Site of analysis matters - Ongoing complete response to Nivolumab in a patient with HIV/HPV related metastatic anal cancer and *MLH1* mutation

**DOI:** 10.18632/oncotarget.28274

**Published:** 2022-09-14

**Authors:** Melanie Demes, Ursula Pession, Jan Jeroch, Falko Schulze, Katrin Eichler, Daniel Martin, Peter Wild, Oliver Waidmann

**Affiliations:** ^1^Dr. Senckenbergisches Institut für Pathologie, Universitätsklinikum Frankfurt, Frankfurt 60590, Germany; ^2^Universitäres Centrum für Tumorerkrankungen (UCT), Universitätsklinikum Frankfurt, Frankfurt 60590, Germany; ^3^Klinik für Allgemein- und Viszeralchirurgie, Universitätsklinikum Frankfurt, Frankfurt 60590, Germany; ^4^Institut für Diagnostische und Interventionelle Radiologie, Universitätsklinikum Frankfurt, Frankfurt 60590, Germany; ^5^Klinik für Strahlentherapie und Onkologie, Universitätsklinikum Frankfurt, Frankfurt 60590, Germany; ^6^Medizinische Klinik 1, Schwerpunkte Gastroenterologie und Hepatologie, Universitätsklinikum Frankfurt, Frankfurt 60590, Germany; ^7^Centrum für Hämatologie und Onkologie, Frankfurt 60389, Germany

**Keywords:** anal cancer, microsatellite instability, immunotherapy, high-throughput nucleotide sequencing, nivolumab

## Abstract

Anal cancer is a rare disease with increasing incidence. In patients with locally recurrent or metastatic disease which cannot be treated with chemoradiotherapy or salvage surgery systemic first-line chemotherapy with carboplatin and paclitaxel is standard of care. For patients who progress after first-line therapy and are still eligible for second-line therapy Programmed cell death protein 1 (PD-1) antibodies are potential therapeutic options. However, prediction of response to immunotherapy is still challenging including anal cancer. We report here to our knowledge the first anal cancer case with microsatellite instability (MSI) due to *MLH1* mutation and a deep and ongoing response to Nivolumab treatment. Namely, thorough analysis of the primary tumor as well as metastatic sites by next generation sequencing (NGS) revealed that MSI was formally only found in the metastatic sites but not in the primary tumor. Concomitantly, tumor mutational burden (TMB) was higher in the metastatic site than in the primary tumor. Therefore, we conclude that all anal cancer patients should be tested for MSI and whenever possible molecular analysis should be performed rather from metastatic sites than from the primary tumor.

## INTRODUCTION

Anal cancer (AC) is a rare, but rising disease with an annual incidence of less than 2 cases per 100,000 inhabitants [1]. The main risk factor for AC development is HPV infection and most common histological type of anal cancer is squamous cell type [2]. In HIV infected males relatively high numbers of AC cases are found [3]. In patients with earlier stages chemoradiotherapy is the standard of care [4, 5]. With a local failure rate of 15% after chemoradiotherapy, salvage surgery is the only chance of cure in patients with persistent or recurrent AC. Overall distant metastasis occurs in 10–20% of patients [5]. There has not been any standard chemotherapy regimen for decades [5]. Recently, convincing data from a randomized phase 2 trial established the combination Carboplatin/Paclitaxel as new standard of care for first-line palliative chemotherapy [6]. At note, there is only little data on second-line chemotherapy treatment in AC patients [7]. For patients with advanced AC two prospective clinical trials showed promising activity of the Programmed cell death protein-1 (PD-1) directed monoclonal antibodies Pembrolizumab and Nivolumab, respectively [8, 9]. However, only two HIV infected patients were treated with Nivolumab in the NCI9673 trial with one of the two treated patients showing response to treatment [9]. Altogether, there is little data on PD-1 treatment in HIV infected patients. Recently, a retrospective analysis of HIV infected veterans treated with nivolumab mainly for lung cancer was published [10]. The efficacy of nivolumab was similar to non-infected patients. However, immune-related adverse events were relatively frequent [10], whereas a systemic review did not find new safety signal [11]. Response prediction to PD-1 directed therapy is still challenging and clinically mostly performed by testing for microsatellite instability (MSI) or DNA mismatch repair (MMR) deficiency [12]. We report a case of an HIV infected patient with anal cancer, MSI high status, a high mutation frequency regarding tumor mutational burden (TMB) and an ongoing response to Nivolumab.

## RESULTS

**Figure 1:**
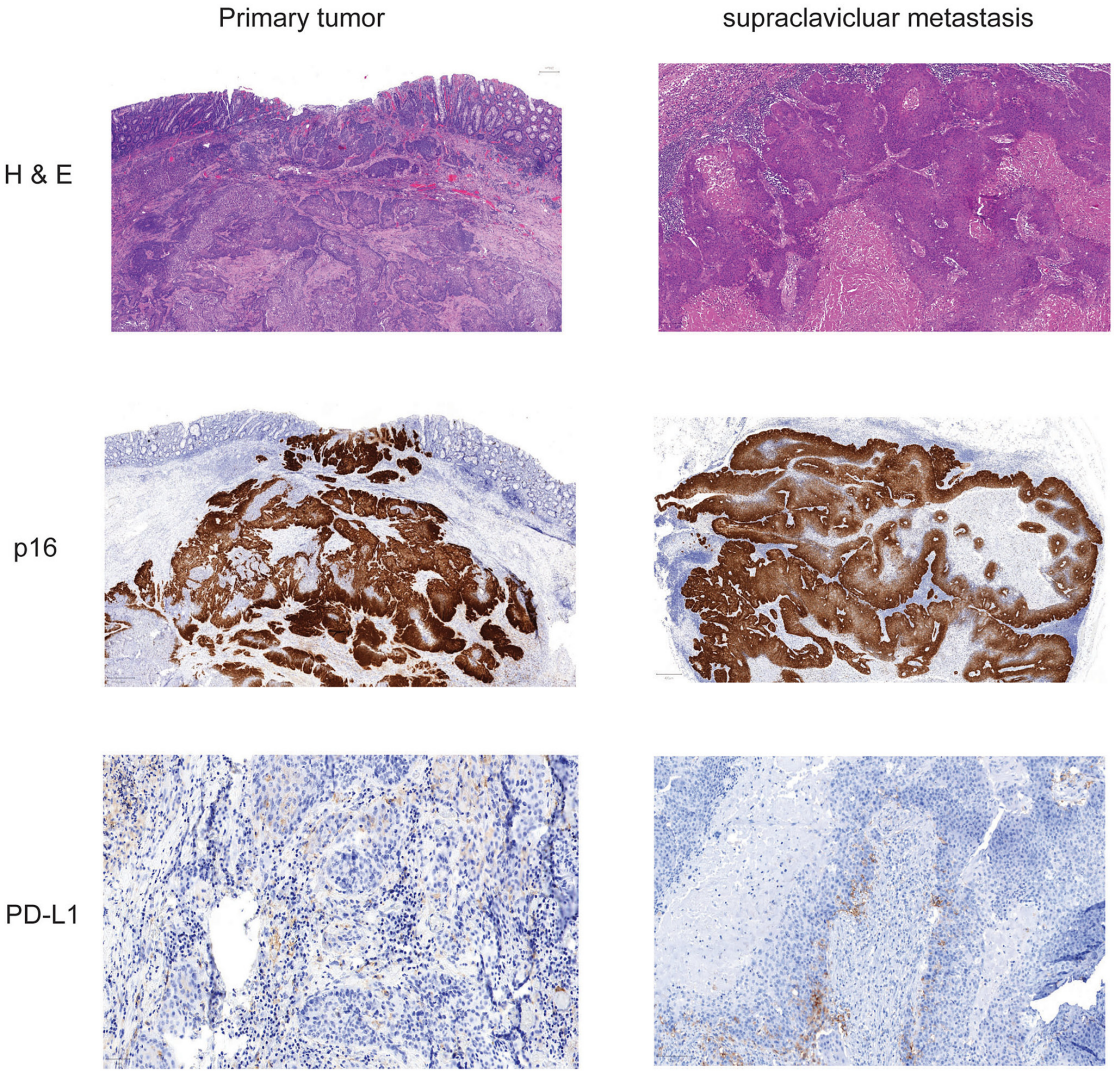
Histochemistry and immunohistochemistry of tumor tissue of primary cancer and lymph node metastasis. Slides from formalin fixed paraffin embedded tissue samples form primary tumor and supraclavicular metastasis were stained for Hematoxylin-eosin, p16 and PD-L1.

**Figure 2:**
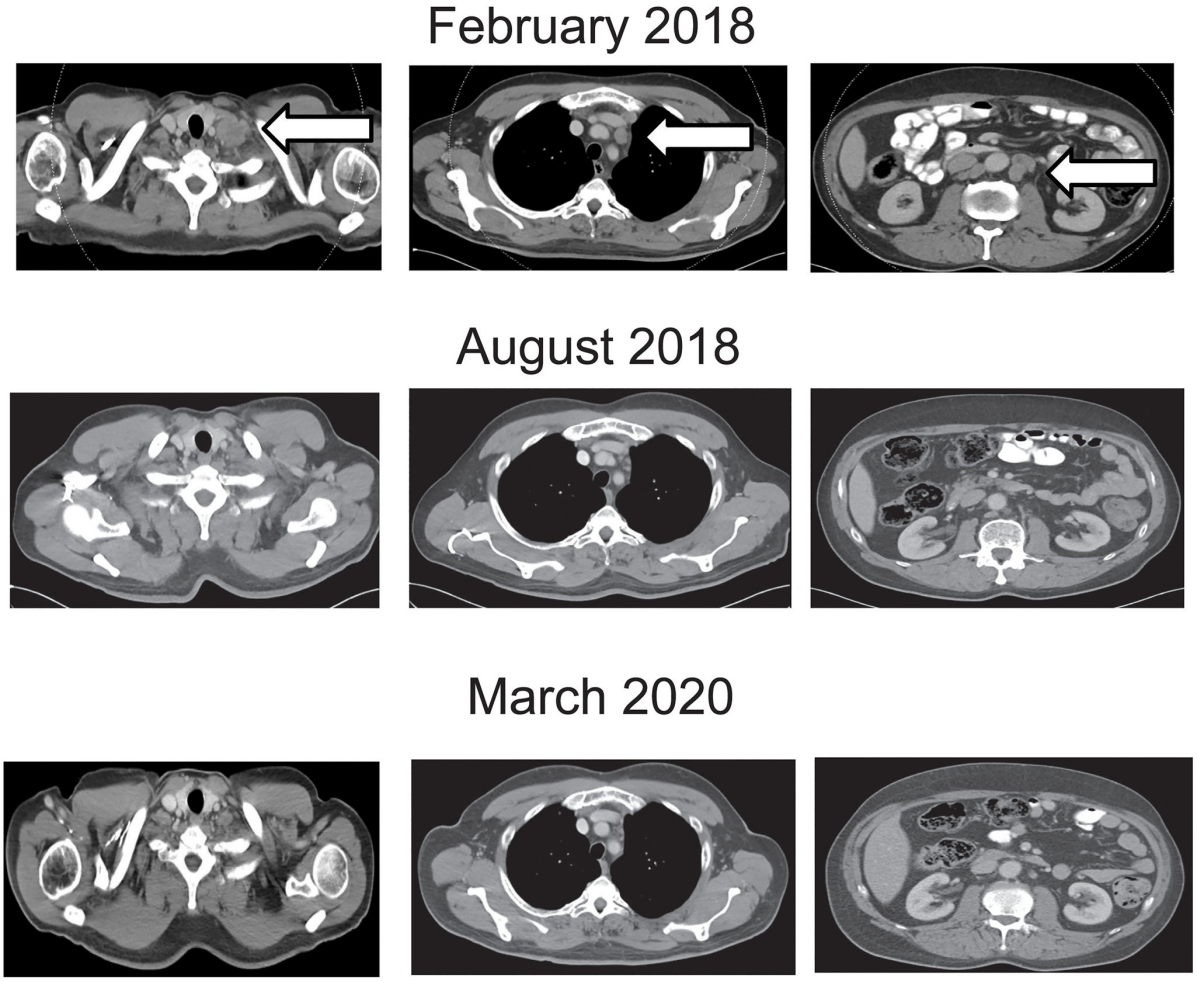
Contrast enhanced computed tomography of the chest and abdomen at beginning, during and at the end of nivolumab treatment. Representative lymph node metastases are shown before, during at end of treatment. Arrows show the metastatic sites before start of treatment.

A 53 years old HIV infected male patient was diagnosed with AC in September 2015. The patient had been diagnosed with HIV 6 years before, in 2009. At first presentation he was on highly active antiretroviral therapy with Dolutegravir and Emtricitabin/Tenofovir. In initial staging there was no sign for distant metastasis but unilateral inguinal and iliac lymph node enlargement was found corresponding to a clinical stage, cT2 cN2 G2. He underwent definitive chemoradiotherapy of the tumor, the involved and elective lymph nodes with a total dose of 59.4 Gy. The patient received fluorouracil 800 mg/m² per day on days 1–5 (week 1) and days 29–33 (week 5) by continuous 24 h intravenous infusion with radiotherapy, and 10 mg/m² of mitomycin as an intravenous bolus on day 1 (week 1) and day 29 (week 5). In May 2016, 17 months after the initial diagnosis, the patient presented with local recurrence. He underwent salvage surgery with abdominoperineal resection (APR) in June 2016. Pathological examination of the resected tissue showed R1 resection (rpT1 ryN0 (0/13) L0 V0 Pn1, R1). Three months later the patient reported a node in the right groin, which was resected and histopathology revealed metastasis of AC. Additional therapy was not performed. Four months later, in February 2017, restaging with computed tomography scans found multiple lymph node metastases in the retroperitoneum up to the left supraclavicular area. Excision of a left supraclavicular lymph node showed metastasis of the AC. Immunohistochemistry found 2% of the tumor cells expressing PD-L1. (Figure 1). First-line chemotherapy with Cisplatin 60 mg/m² (d1) and Capecitabine 2500 mg/m² (d1-14) every three weeks was started in March 2017. First staging after three months showed stable disease, therefore chemotherapy was continued. After additional three months of therapy, in September 2017 progression of lymph node metastasis was detected. Chemotherapy was escalated by adding Docetaxel 75 mg/m² (d1) to the chemotherapy regimen with Cisplatin 60 mg/m² (d1) and Capecitabine 2500 mg/m² (d1-14) every three weeks. Next staging in January 2018 showed again progressive disease. Given the promising data from the NCI9673 trial [9], treatment with Nivolumab 3 mg/kg every 2 weeks was initiated in February 2018. Staging with contrast-enhanced computed tomography after three months in May 2018 showed regression of lymph node metastasis (Figure 2). Treatment was continued and next staging in August 2018 revealed partial response, and complete response in February 2019. Treatment was continued until February 2020 and stopped thereafter with ongoing complete response in March 2020 (Figure 2). Patient showed ongoing response to therapy up to February 2021. During chemotherapy with Cisplatin, Capecitabine and Docetaxel CD4 counts decreased to 87/μl. After the end of chemotherapy and after initiation of immunotherapy (IT) CD4 cells raised to 171/μl and lately to 264/μl. HIV 1-RNA has not been detectable during all treatment courses.

## DISCUSSION

AC is a rare disease with increasing rates of new diagnoses [1]. Especially male HIV infected patients have a relatively high risk for AC development [3]. The predominant number of AC in patients with HIV infection are HPV related [3]. Cancers associated with viral infections often show an inflammatory microenvironment [13]. In patients with such virus-associated cancers higher response rates to IT are found compared to the response rates in patients with cancers not associated with viral infections [14]. The potential of IT to induce responses and disease stabilization in AC patients was shown in the NCI9673 trial [9]. However, only 2 of 37 treated patients were HIV positive and only one of the two patients showed a response [9]. Overall, patients with response had higher PD-L1 levels in the tumors and higher concentration of CD8 T cells in the surrounding tissue [9]. In our patient strong p16 expression in the tumor cells was seen fitting to HPV infection. Additionally, PD-L1 expression was also found in the tumor cells, although in a relative low proportion of 2%. In the KEYNOTE-028 study, patients with PD-L1 expression of ≥1% including both neoplastic cells and contiguous mononuclear inflammatory cells, received Pembrolizumab 10mg/kg body weight every two weeks for up to two years [8]. The response rate was comparable with 17%. However, HIV infected patients were excluded from the trial (NCT02054806). Generally, HIV infection is associated with increased expression of PD1 on T-cells leading to impairment of T cell activation [15]. PD-L1 blockade modulates and restores cytokine production and leads to proliferation of HIV specific CD4 T-cells [16]. In our case we observed a rising count of CD4 positive cells by persistent negative HIV 1-RNA status during Nivolumab therapy (data not shown). Safety and efficacy of PD-1 treatment in real life has recently been reported in a French cohort of HIV infected patients [17].

Prediction of response to IT is still challenging. Microsatellite instability (MSI) and to a somewhat lesser extent TMB are predictors of response to PD1/PD-L1 directed therapy [18]. Namely, MSI is rare but high TMB is frequently found in AC [18]. To our knowledge no patient case with HIV associated AC, MSI and ongoing response has been published. The mismatch repair mechanism corrects mismatches generated during DNA replication and recombination and therefore maintains genomic stability. Mismatch repair (MMR) proteins/genes may show a loss of function characterizing a MMR-deficient (MMR-D) tumor. In this particular case MLH1 and PMS2 expression was lost at all tumor sites. These specific proteins were still expressed in the tumor surrounding tissue indicating an somatic event. We also found the mutation of *MLH1* in the primary tumor as well as in the metastatic sites. The allele frequency of detected alterations rather indicates an somatic event, too. Loss of PMS2 expression can be also caused by MLH1 mutation or MLH1 promotor hypermethylation. Generally MLH1 and PMS2 form heterodimers that may repair damages in the DNA. According to some studies, human immunodeficiency virus infection was associated with a microsatellite unstable tumor. Thus a study reveals a shift to MLH1 loss in gastric cancer with high HIV prevalence [19–21].

*PIK3CA* mutations were detected in the metastatic and primary tumor. Oncogenic *PIK3CA* mutations contribute to tumorigenesis by activating AKT signaling to decrease apoptosis and increase tumor invasion. Distinct studies have reported that *PIK3CA* mutation is more commonly mutated in the MSI molecular subgroup. According to the literature *PIK3CA* mutations has an independent prognostic value for patients who underwent recurrence. This subgroup may potentially benefit from adjuvant treatment and targeted therapies with PI3K/Akt/mTor inhibitors [22–24].

No mutational event was detected in the genes *RAS* and *BRAF*.

A high concordance rate between primary cancer and metastatic disease concerning driver mutations such as *RAS* or *BRAF* but also MSI has been reported from colorectal cancer [25–27]. A clinically relevant proportion of unstable MSI sites and also a high TMB were only found in the metastatic sites. As TMB is predictor for tumor response in different cancer entities including lung cancer and AC [28–30]. Response prediction to PD-1 directed therapy is still challenging and clinically mostly performed by testing for microsatellite instability (MSI) or DNA mismatch repair (MMR) deficiency [12]. This case report represents an HIV infected patient with anal cancer, MSI high status, a high mutation frequency regarding tumor mutational burden (TMB) and an ongoing response to Nivolumab. According to our results, we propose to assess mutational status in tissue from metastasis rather than from the primary site when additional molecular analyses are performed for treatment decisions.

## MATERIALS AND METHODS

Immunohistochemical staining of the PD-L1 (clone ZR3), p16 (clone INK4A) as well as mismatch repair (MMR) proteins MLH1 (clone BS29), MSH2 (clone G219-1129), MSH6 (clone SP93) and PMS2 (clone EP51) was done using the OMNIS (Agilent) automated advanced staining technique. To determine the tumor area of interest and corresponding tumor cell content the tissue samples were stained with hematoxylin/eosin by the Sakura Tissue-Tek Prisma^®^ Plus and Tissue-Tek Film^®^. An experienced pathologist assessed the slides.

For molecular characterization of a 523-gene panel (TSO500, Illumina, San Diego, California, USA), paraffin-embedded tissue sections were macrodissected from tumor for DNA and RNA extraction. The purification of DNA and RNA from the formalin-fixed paraffin embedded (FFPE) tissue samples was performed using the QIAamp FFPE Tissue Kit (QIAGEN) and the Maxwell^®^ RSC FFPE DNA Kit (Promega Corporation, Madison, Wisconsin, USA), respectively. Further preparation and the instrument run were performed according to manufacturer’s protocol. The concentration of DNA and RNA was measured with the Qubit 4 Fluorometer (Invitrogen, Thermo Fisher, Waltham, Massachusetts, USA). For the library preparation the hybrid capture-based TSO 500 library preparation kit (Illumina) were finally sequenced using V2 sequencing reagent kits on a NextSeq550 platform (Illumina) according to manufacturer recommendations.

Data analysis was performed using the analysis BaseSpace TSO 500 Assessment App (Illumina). Molecular Health was used as interpretation software.

A variant allele frequency (VAF) >5% and a total read depth of >50X were filtered for analysis. For MSI, 130 MSI loci sites were algorithmic analyzed by calculating a quantitative score. The performance of TMB detection is set by analyzing SNVs and indels in the coding regions reading somatic alterations. The TMB score is defined as the number of eligible somatic mutations per Mb (targeted region defined as high confidence regions with >50× coverage).

### Histological and molecular analysis

**Figure 3:**
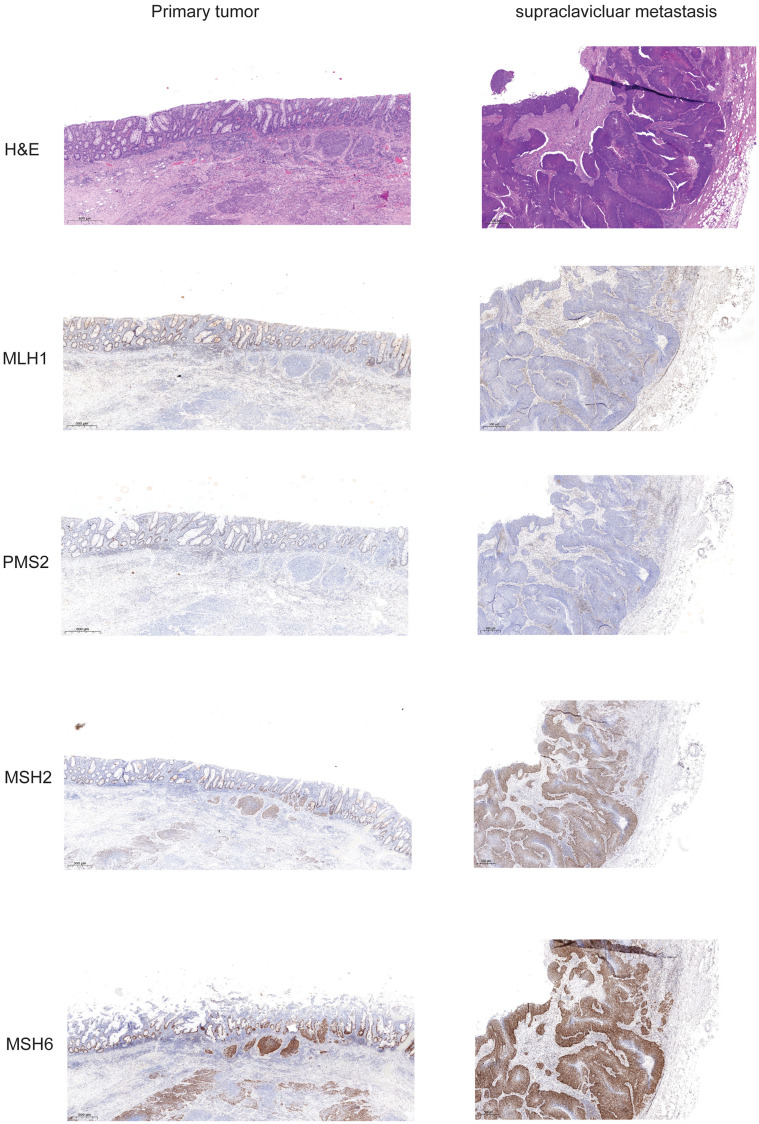
Expression of mismatch repair proteins in primary tumor and supraclavicular metastasis. Slides from formalin fixed paraffin embedded tissue samples form primary tumor and supraclavicular metastasis were stained for Hematoxylin-eosin, and the mismatch repair proteins MLH1, PMS2, MSH2, and MSH6.

FFPE material was accessible from the resected primary tumor, inguinal metastasis as well as from a supraclavicular lymph node metastasis. Staining for H&E, p16 and PD-L1 of different tumors was performed (Figure 1). p16 expression was detected at the primary site and the metastasis and PD-L1 expression was found in 2% of the tumor cells indicating a TPS Score of 2%. As the patient showed impressive response to Nivolumab we performed additional staining for MMR proteins. At both analyzed tumor probes loss of MLH1 and PMS2 was found, but MLH1 and PMS2 were still expressed in tumor surrounding tissue (Figure 3). However, only the two biopsies of the metastatic site but not the tissue from the primary cancer showed more than 20% of unstable MSI sites (Table 1A). For further work-up NGS analysis of three tumor site was performed. *MLH1* mutation (c.588dupA) could be detected with an allelic frequency (AF) ranging from 40% to 50% at all three tumor sites. Additionally, all three tumor sites showed a *PIK3CA* mutation (p.E545K) (AF ranging from 45% to 48%). It is known, that activating mutations in *PIK3CA* drive anal carcinogenesis and that the PI3K/mTOR pathway is a relevant target for therapeutic intervention. TMB was calculated from the analysis from the different sites. Namely only the metastatic sites showed a high TMB with the defined TMB-H threshold of > 20 mutations per megabase (Table 1B).

**Table 1A:** Microsatellite instability (MSI), cutoff (percent of unstable MSI sites) = 20%

	Primary tumor	Supraclavicular metastasis	Inguinal metastasis
Unstable MSI sites	155	117	115
Unstable MSI sites (total)	17	29	25
Percent unstable MSI sites	14.78	24.79	21.74
MSI status	stable	unstable	unstable

**Table 1B:** Tumor mutational burden (TMB), coding target region size 1.31 MB

	Primary tumor	Supraclavicular metastasis	Inguinal metastasis
Non-synonymous SNVs	15 (11.43 Mut/Mb)	29 (22.10 Mut/Mb)	35 (26.67 Mut/Mb)
synonymous SNVs	4 (3.05 Mut/Mb)	13 (9.91 Mut/Mb)	13 (9.91 Mut/Mb)
Deletions, insertions, indels	4 (3.05 Mut/Mb)	9 (6.86 Mut/Mb)	12 (9.15 Mut/Mb)
TMB based on selected variant types (Mut/Mb)	17.53 (TMB-L/TMB-H)	38.87 (TMB-H)	45.73 (TMB-H)

Defined TMB-L threshold TMB (Mut/Mb) < 5

Defined TMB-H threshold TMB (Mut/Mb) > 20

### Patient update

Given the promising data from the NCI9673 trial [9], treatment with Nivolumab 3 mg/kg every 2 weeks was initiated in February 2018. Staging with contrast-enhanced computed tomography after three months in May 2018 showed regression of lymph node metastasis (Figure 2). Treatment was continued and next staging in August 2018 revealed partial response, and complete response in February 2019. Treatment was continued until February 2020 and stopped thereafter with ongoing complete response in March 2020 (Figure 2). Patient showed ongoing response to therapy up to February 2021. During chemotherapy with Cisplatin, Capecitabine and Docetaxel CD4 counts decreased to 87/μl. After the end of chemotherapy and after initiation of immunotherapy (IT) CD4 cells raised to 171/μl and lately to 264/μl. HIV 1-RNA has not been detectable during all treatment courses.
